# Variation in Hepatitis C services may lead to inequity of heath-care provision: a survey of the organisation and delivery of services in the United Kingdom

**DOI:** 10.1186/1471-2458-6-3

**Published:** 2006-01-10

**Authors:** Julie Parkes, Paul Roderick, Bethan Bennett-Lloyd, William Rosenberg

**Affiliations:** 1 Public Health Sciences & Medical Statistics (805) Level C, University of Southampton, Southampton General Hospital, Tremona Road, Southampton UK; 2 Wellcome Trust Clinical Research Facility, Southampton General Hospital, Tremona Road, Southampton UK; 3 Southampton Liver Group, University of Southampton, Southampton General Hospital, Tremona Road, Southampton UK

## Abstract

**Background:**

Chronic hepatitis C infection (CHC) is a major healthcare problem. Effective anti-viral therapy is available. To maximise population effectiveness, co-ordinated services for detection and management of patients with CHC are required. There is a need to determine patterns of healthcare delivery to plan improvements. A study was conducted to determine workload, configuration and care processes of current UK services available to manage patients with CHC.

**Methods:**

A cross-sectional questionnaire survey of consultant members of British Association for the Study of the Liver (n = 53), Infectious Disease consultants (n = 43), and a 1 in 5 sample of Genito-Urinary Medicine (n = 48) and gastroenterologists (n = 200).

**Results:**

Response rate was 70%. 40% of respondents provided a comprehensive service (included treatment and follow-up): speciality of clinical leads identified as Hepatology (37%); Gastroenterology (47%); and Infectious Disease (16%). The estimated number of patients managed by respondents was about 23,000 with an upward trend over the previous 3 years. There was variation between comprehensive service providers, including unit size, eligibility criteria for treatment, and drug regimes. Key barriers to quality of care identified were staffing capacity, funding of treatment and patient non-attendance. Most English strategic health authorities had at least one comprehensive service provider.

**Conclusion:**

There was significant variation in all aspects of the patient pathway which may contribute to inequity of health care provision. Services need to be expanded to form geographical clinical networks, and properly resourced to ensure greater uptake and more equitable delivery of services if the future burden of chronic liver disease is to be reduced.

## Background

Chronic Hepatitis C (CHC) is a major cause of serious liver disease[1,2]. Effective viral eradication therapy using combinations of anti-viral agents is available [3,4] and guidance for their use in the UK has been issued [5,6]. Since the screening of blood donors for hepatitis C (HCV) was introduced, the main risk groups are ex and current injecting drug-users (IDUs), who are hard to reach by health care services.

National Strategies for Hepatitis C in the UK have been published [7-9]. These recognize the need for a systematic approach to the identification, testing, referral, selection for treatment and follow-up of HCV positive patients. They recommend the establishment of managed clinical networks for CHC -managed clinical networks may be defined as linked groups of health professionals working in a coordinated manner across organisations and structural boundaries, with a common strategic agenda to promote health improvement and reduce health inequalities for a given population thus maximising shared resources in a coordinated way[10]. They also suggested a national survey of current practice and service configuration to establish baseline information upon which to plan the future services for patients with CHC. This study reports on the findings of such a survey.

## Methods

The four clinical specialties most likely to manage patients with Hepatitis C – Gastroenterology (GI), Genito-Urinary Medicine (GUM), Hepatology, and Infectious Diseases (ID) were targeted by postal questionnaire sent out in 2002. The sample was drawn from memberships lists supplied by the respective professional organisations.

All consultant/equivalent grade members of the British Association for the Study of the Liver (BASL) were deemed to be "hepatologists" by virtue of expressing a particular interest in liver medicine.

A list of Infectious Disease Units in the UK was provided and a consultant Infectious Disease (ID) Physician from each hospital was invited to participate.

Gastroenterologists at consultant grade were identified by membership of British Society of Gastroenterology and a 1:5 random sample was taken, stratified by Health Region. A similar approach was used to sample Genito-Urinary Medicine consultants identified by membership of the Association of Genito-Urinary Physicians.

An expert steering group was consulted in the design of a self-completed questionnaire. This was piloted on 20 physicians randomly sampled from each of the four clinical specialties, the comments from the pilot informing the final questionnaire that was sent out to the whole sample described above (see Additional file 1). Subjects who did not have any role in the management of HCV infection were asked to provide the name of the lead clinician who fulfilled this role for their catchment area, and a questionnaire was then sent on to these individuals. Those who did not respond were contacted once by telephone in order to improve the response rate.

Respondents were asked to classify their service according to the three following descriptors: "providing diagnostic and investigative services but **not **treatment (diagnostic investigative provider – DIP)"; "providing diagnostic testing, investigations, treatment and follow-up of patients (comprehensive service provider-CSP)"; and "no role in the management of hepatitis C infection". Several questions asked for a response in terms of the percentage of patients; these used groupings 0–4%, 5–9%, 10–24%, 25–49%, 50–74%, 75+%.

The information derived from returned questionnaires was analysed using SPSS statistical package (SPSS for Windows 11). Standard statistics for comparing groups were used.

## Results

### Response rate

The response rate for the initial survey was 71% (203/287). A further 57 clinicians were identified as providers of a comprehensive service by respondents who did not themselves provide such a service. Of these, 65% (37/57) responded giving a total response rate of 70% (240/344).

40% (97) of respondents were comprehensive service providers (CSPs). 46 (47%) of the CSPs had a gastroenterology lead, 36 (37%) a hepatology lead, and 15 (16%) an ID lead. GUM physicians provided a service up to DIP 26 (26%), but none were CSPs.

Almost three-quarters of hepatologists and half of ID physicians were CSPs. The majority of gastroenterologists (63%) provided either no role or a DIP service for patients with HCV (see Table 1).

DIPs were mostly sited in small or medium sized district hospitals with an urban/mixed urban catchment population. Most looked after a small number of patients with HCV infection, with 52% diagnosing less than 10 patients with HCV (2001) and 82% diagnosing less than 20 patients.

More of the CSPs covered catchment populations of over 1 million (15%) compared to DIP (8%), with more of the largest catchment areas being served by hepatologists (p = 0.006). CSPs saw a larger new caseload with 50% of respondents seeing >40 patients a year.

The total estimated prevalent population of patients with CHC managed by the responding CSPs was 22,100, of which 50% were managed by hepatologists, and 25% each by ID physicians and gastroenterologists. The number of prevalent patients varied between CSPs, the median number was in the range 100–500 patients; at the extremes nine CSPs managed under 20 cases in total and three CSPs were managing over 1000 patients in total. (There may be some uncertainty in who is included in these figures, as the questionnaire asked for patient numbers "*currently under your care*" which may include those defaulting and not yet discharged back to Primary Care). One third of CSPs saw >75 new cases of HCV in 2001.

### Identification and referral of HCV patients (Table 2)

For half of the CSPs the diagnosis of HCV infection was already made at the time of referral. Of patients referred with an established diagnosis, 41% originated in primary care, 24% in drug and alcohol services, 14% in prison healthcare and 14% from GUM physicians.

Of those referrals where the CSP unit established the diagnosis of Hepatitis C, most came from primary care (73%), other clinical specialties (mainly gastroenterology (17%) and ID (12%)), and drug and alcohol agencies (14%).

50% (48) of CSPs were aware of active case finding in their catchment populations, with 19% (9) of case finding being performed in accordance with a written policy.

Most case finding was performed in patients with abnormal liver tests, or with identified risk factors such as IDU.

24% (22) of CSPs had outreach services, most commonly in prisons (17) or drug and Alcohol services (9).

Default rates reported for the initial out-patient appointment were high, with the median per CSP being 10–24%. There was no association between the default rate and size of unit (p = 0.19).

### Patient management

Co-ordinated management strategies for patients with HCV were reported by 45%% (44) of CSPs, with formal collaboration between CSP and other services. The most common formal links were between the CSP and Drug and Alcohol Teams (n = 37), GUM services (n = 30), prisons (n = 22), primary care (n = 20) and homeless units (n = 14). Other links included asylum seekers (2), other medical specialities (5) and a renal dialysis unit (1).

57% of CSPs had a hospital based multidisciplinary team (MDT) that met regularly to discuss the management of HCV patients. The composition varied but most commonly included the consultant from the lead clinical specialty, specialist nurse, pathologist, radiologist and DAT representative. Only three MDTs had a patient representative.

The larger units led by Hepatologists and ID Physicians had more access to specialist nurses, who played a key role in starting treatment and in monitoring patients.

### Drug treatment

10–24% (median) of new patients was considered eligible for anti-viral drug treatment. Table 3 shows the factors used to determine eligibility. Over 90% of responding CSPs used severity of hepatitis, more than 60% of respondents used age, and 90% used co-morbidity. The most important single reason reported for ineligibility, by over 60% of CSPs, was ongoing illicit drug use. The most common reason given for why patients refused treatment was concern over side effects (see Table 3).

24% of CSPs reported that over 90% of the eligible patients were treated, with 50% reporting that over 75% of eligible patients were treated (median range 50%–74%). The majority were not treated in clinical trials.

35% of CSPs offered treatment to patients with mild hepatitis -of these 95% offered treatment to patients with symptoms and 49% to patients who were asymptomatic.

Virtually all of the respondents definitely offered treatment to patients with moderate or severe hepatitis /cirrhosis (Child-Pugh A), 48% (38) (with 9% (7) "maybe treat") also treated those with Child-Pugh B, and 14% (11) treated patients with Child Pugh C cirrhosis. 16% (and 8% "maybe") offered antiviral treatment for those awaiting transplantation. In the majority of cases there was no link between the CSP treating HCV related end-stage liver disease with anti-virals and whether they were transplant centres or had close collaboration with transplant centres. (Child Pugh is a clinical score to evaluate prognosis in cirrhosis using bilirubin, albumin, INR, ascites and encephalopathy which are awarded points depending on values., and allocated to three categories which indicate increasing severity of disease Class A (5–6 points) Class B (7–9 points) Class C (10–15 points). Class A has a better prognosis than B, which in turn has a better prognosis than class C).

Two thirds (66%) of respondents currently used interferon and ribavirin combination therapy and a majority (59%) were using pegylated interferons. Those CSPs who used pegylated interferon and ribavirin used it in 50–74% of treated patients (median), and those CSPs who used interferon and ribavirin used this regime in 25–49% of treated patients (median) (see Table 4). Interferon and pegylated interferon alone were used in the minority of patients treated by CSPs (0–5%), Just over half (52%) of CSPs reported that they used printed guidelines for dose reduction and stopping therapy and 35% performed post treatment liver biopsy.

The median rate of adherence to treatment reported was 75–90%, with the main reasons for stopping treatment being 'patient initiated' and 'side effects'. There were no significant differences in the percentage of CSPs reporting higher levels of adherence by clinical specialty, presence of a specialist nurse, or size of unit though the study is underpowered to detect any differences.

For those patients not receiving treatment there was variation in practice for the follow-up of patients with different degrees of severity of hepatitis. The median follow-up for patients not on treatment with mild hepatitis was annually (66%), for moderate hepatitis it was every 6 months (55%), and for severe hepatitis and cirrhosis it was every 3 months (47%).

The majority of CSPs (62%) had a database to record information about patients with HCV and another 12% were in the process of establishing a database. This varied by clinical speciality from 57% for GI led units, 88% for hepatologist and 93% for ID. 15 data systems were in paper format and 57 were electronic (some units maintained both forms). There was concordance of minimum data items collected.

### Barriers to providing a high quality service for patients with Hepatitis C

82% of CSPs reported barriers in the management of patients with Hepatitis C. The main factors are shown in Table 5. Funding, staff capacity and patient non-attendance were the most common. Biopsy waiting times showed some differences between specialities, with 17 hepatologists and 4 ID physicians agreeing/strongly agreeing that biopsy times are a barrier to care, and 6 Hepatologists and 9 ID physicians disagreeing.

### Geographical distribution of services

**Figure 1:**
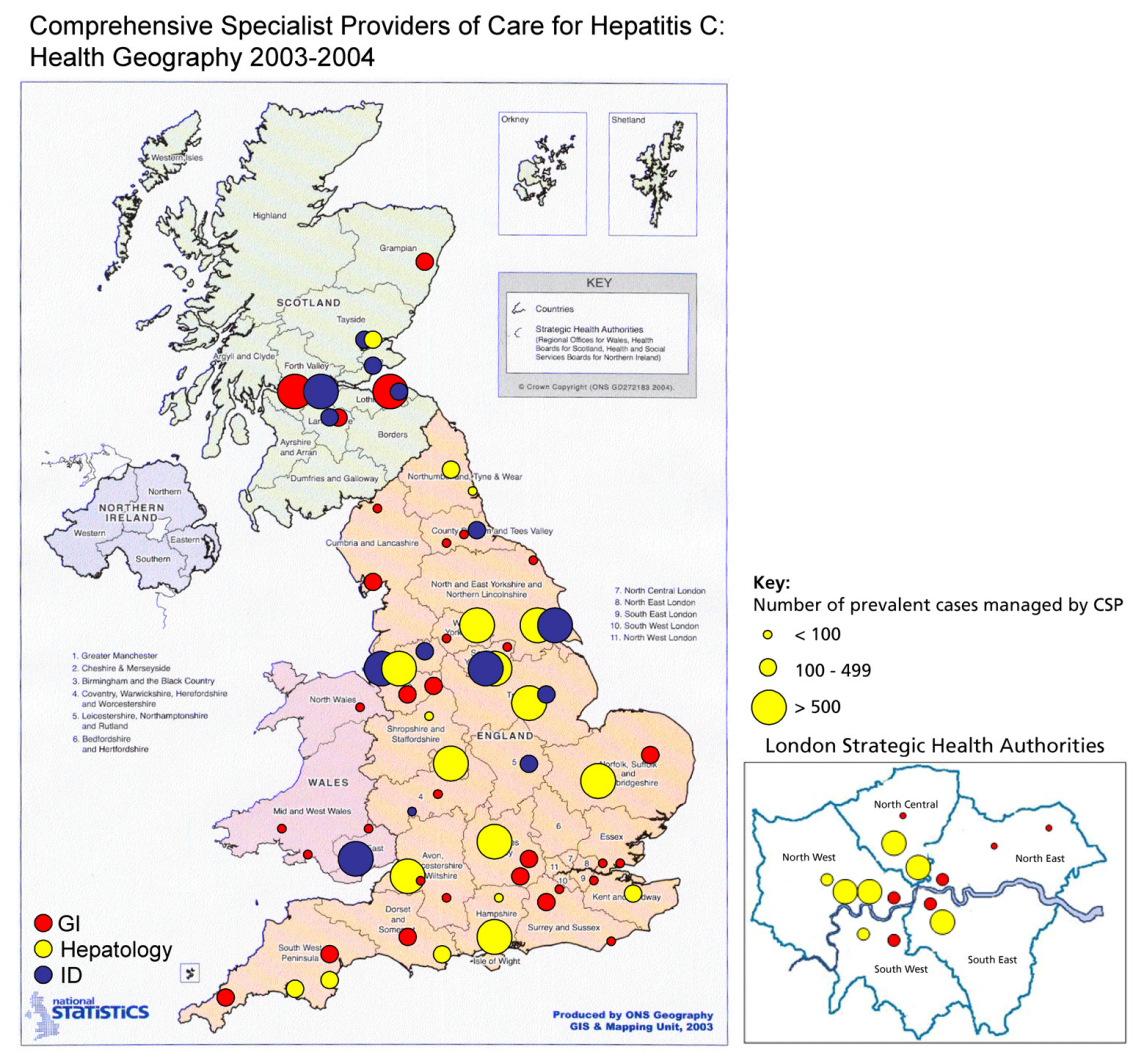
Distribution of comprehensive service providers in the UK by health geography (SHA, Health Board), numbers of prevalent patients with chronic hepatitis C managed per provider, and clinical lead speciality.

In England, 27 out of 28 Strategic Health Authorities (SHAs) had a CSP within their boundary (Figure 1). The size of the CSP and the clinical discipline of the lead varied, with some SHAs having a few smaller centres, and others a single large provider. ID led units were concentrated in the North/Midlands of England and Scotland. Almost one third of the SHAs (32%) did not have a CSP led by a hepatologist and one quarter did not have a hepatologist within the SHA. Over half of the SHAs had ≥ 5 prisons, and there was no obvious relationship between the presence of CSPs and the number of prisons.

In Scotland, there were CSPs in 6 out of 15 health boards, with the larger centres being in Glasgow, Edinburgh and Dundee, and they were mostly led by ID physicians or gastroenterologists. In Wales five CSPs were identified, one of which was led by an ID physician and four by gastroenterologists.

## Discussion

This survey included a national representative sample of consultant clinicians managing patients with Hepatitis C. It had a high response rate with wide geographical spread of respondents. By conducting a two stage process we think we captured most CSPs. The study revealed variations in structure and process at all stages of the patient pathway, such as size of unit, clinical lead, eligibility criteria for treatment, and drug regimes used. Barriers to care were identified which contributed to difficulties experienced by services in coping with the present burden of patients in the hospital system. Such variation in healthcare provision may contribute to inequity of care for people with Hepatitis C. Having provided an analysis of the provision of Hepatitis C services in the UK, this survey can be used as a base for future planning and development, and the urgent need to address inequalities.

This survey found that the majority of patients within CSPs are not receiving anti-viral treatment. This is supported by industry figures of approximately 10,000 UK patients treated so far (personal communication Schering Plough, Roche)[11]. This contrasts with practice in Europe. 56% of positive subjects in France now know their serological status and 75,000 have been treated. 100,000 have been diagnosed in Germany, with 20–30,000 treated. Italy has treated approximately 100,000 patients, one third of diagnoses. In France through a wider screening policy (including prisoners, antenatal clinics, and social security medicals), reimbursement of private laboratories, and greater awareness, testing increased by 26% and 1.2 million tests were performed in France between 2000 and 2002. Testing is freely available anonymously in French universities, town centres and hospital family planning clinics in addition to prisons and drugs clinics. Germany has a similar screening strategy (personal communication Bethan Bennett Lloyd). There is no formal screening programme in the UK except for blood donors, although the Hepatitis C Action Plan plans a case finding strategy for named high risk groups.

NICE guidance in the UK recommends that interferon (pegylated and non pegylated) and ribavirin be used in moderate and severe CHC. A.European consensus (1999) statement recommended that moderate/severe necroinflammation and/or fibrosis be treated, and in the USA treatment of moderate and severe disease with pegylated interferon and ribavirin is recommended [12-14]. Despite the national guidance many CSPs found that funding for treatment was a barrier to care, leading to a persistence of postcode prescribing with regional variation in access to treatment. The survey showed that there was a high attrition rate of people who could potentially benefit from specialist services, at the point of identification of high risk groups, diagnosis, referral to specialist services, defaulting from clinic, determination of eligibility for treatment and those finally receiving treatment. Further elaboration on the reasons for this attrition may contribute to more effective strategies aimed at increasing the number of people potentially eligible for treatment being seen by specialist services.

**Figure 2:**
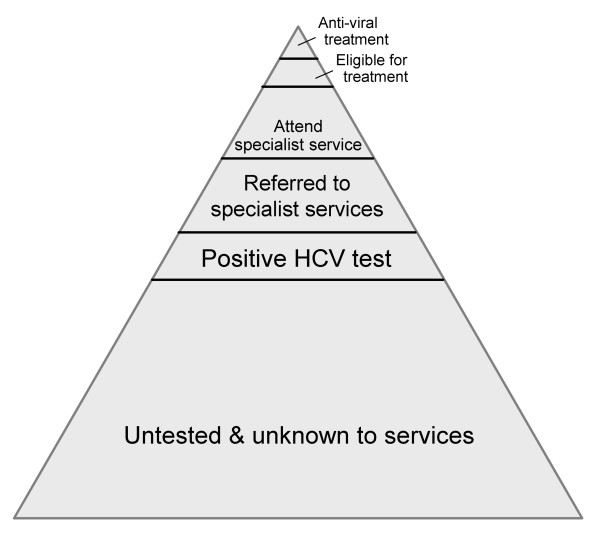
The disease iceberg for Hepatitis C.

The true prevalence of CHC in the UK is unknown. Estimates remain vulnerable to the lack of information of the "ever" and current IDU population at risk of CHC, leading to wide ranges around best guesses. The cumulative number of antibody positive HCV tests reported in the UK is 59,000 (1992–2003)[15,16], which suggests that even all those who have a antibody positive test are not being managed by specialist services. Most people with CHC are therefore likely to be unknown to secondary care, and of these there are those who are undetected and unknown to services, those in contact with services but not tested, those who are diagnosed but who are not referred to specialist health services, and those who are referred but do not attend (see figure 2). Best estimates derived by pragmatic modelling of this prevalent population range from 200,000–500,000 [17,18] (unpublished data- submitted for publication Parkes J. et al). By 2020, using existing models, the future burden of cirrhosis due to HCV is estimated to be three times that at present, posing major resource implications for services [19-22].

How can services identified by this study be improved to meet the needs of patients with CHC, and reduce the future burden of liver disease? This survey found that a large number of gastroenterologists were caring for a small number of patients with CHC. However, most patients with CHC were cared for by medium sized units led by hepatologists or ID physicians. The diversity in clinical disciplines leading CSPs may have contributed to the relative lack of co-ordination in care and the low awareness of Hepatitis C in the UK. Recent national initiatives may have gone some way to improve this situation and should be built on to provide a cohesive national service for people with CHC. Models of best practice were identified by this survey (data not presented), the salient feature being coordination and cooperation between the relevant specialities and settings in a locality involved in managing people with CHC.[23] The variation in management strategies including treatment despite national standards of care may also be well served by increased cohesion of services locally and nationally, with the aim to have a consistent high quality evidence based service delivered to patients with CHC no matter where they live.

Staff capacity was identified as a major barrier to care, with all aspects of a CHC service needing additional resources. As the number of patients with advanced liver disease due to CHC grows, this will become ever more pressing with major implications for health care commissioners and providers with respect to recruitment, training and funding of specialised hepatology staff.

Although there has been no co-ordinated policy driving service configuration, there was a CSP in virtually all English SHAs, and in many Welsh and Scottish health districts. These centres provide the potential for building managed clinical networks based on populations of around 1–2 million as recommended by national policies. Such networks could act to ensure national standards and equity of access to services, working together across localities and coordinating services across the country. CSPs should be developed within each network with a critical mass of staff, clear referral guidelines, outreach arrangements, and a unified information system to support these networks and to monitor service performance. The early establishment of a comprehensive computerised IM&T strategy is needed as a matter of some urgency. It is encouraging that many of the CSPs were collecting homogenous data on CHC patients, however there was variation in the computer systems used and many units were unable to put data on to electronic systems due to lack of staff.

Commissioners of HCV services will need to assess the population need in their area, which will be driven predominantly by local IDU prevalence (current and ex), including that within the local prison population [24,25]. Targeting of high risk groups such as IDUs in prisons has been shown to be not systematised, with this survey reporting only 17 CSPs who had collaborative links or outreach clinics in prisons out of a total of 158 UK prisons [26-29]. More effort is needed to access this hard to reach population whilst they are detained, with risk minimisation, case finding and treatment of eligible people. More research is needed to evaluate models of delivery of Hepatitis C services to prisoners, both in prison and after their discharge.

Limitations to this survey include inaccuracies in the professional lists used to sample participants. Whilst this may have led us to miss specialist centres, coverage appears to be good. Hepatology is not yet recognised as a specialty, and we had to assume that those clinicians belonging to national specialist groups have an interest in liver disease and we used this as a definition of hepatologist. The quantitative responses were self -reported and may not have been derived from accurately recorded sources. Using the DIP and CSP classification was imperfect as several DIPs reported fluidity between categories depending on external factors such as resources.

## Conclusion

This survey has examined how individuals are identified, diagnosed and treated. It has shown that there is inequity and variation in the management of people with CHC in the UK, with staffing and funding of treatment as key barriers to care. However, based on existing specialist centres identified by this survey, there are opportunities to provide planned care via managed clinical networks supported by a national information strategy. Urgent investment is needed to improve the identification, referral and management of people with CHC, and to maximize the opportunities to reduce the sequelae of serious liver disease.

## Competing interests

The author(s) declare that they have no competing interests.

## Authors' contributions

JP and BBL designed the questionnaire, carried out the survey

JP performed data analysis and wrote the first draft of the paper

BBL conducted the follow up of non returns, performed data entry, provided administrative support to the study, and contributed to writing the paper

PR provided expert epidemiology and public health input, supervised the work, and contributed to writing the paper

WR conceived of the study, provided clinical expertise, supervised the work and contributed to writing the paper

All authors read and approved the final manuscript.

## Pre-publication history

The pre-publication history for this paper can be accessed here:



## Supplementary Material

Additional File 1 Questionnaire-*For questionnaire used in the national survey*. Click here for file

## Figures and Tables

**Table 1:** Response rates & role in the care of patients with Chronic Hepatitis C by specialty

			Management role by specialty*		
	**Number in survey n (%)**	**Overall responding n (% total)**	**No role n (%)**	**DIP n (%)**	**CSP n (%)**

**GI**	200 (58)	124 (52)	28 (64)	50 (51)	46 (47)
**Hepatology**	53 (15)	48 (19)	3 (7)	9 (9)	36 (37)
**ID**	43 (13)	30 (13)	1 (2)	14 (14)	15 (16)
**GUM**	48 (14)	38 (16)	12 (27)	26 (26)	0 (0)
**Total**	344 (100)	240 (70)	44 (100)	99 (100)	97 (100)

DIP = diagnostic & investigative provide CSP = comprehensive service provider

* e.g. number (%) of management role provided by speciality

**Table 2:** Referral pathways of HCV patients to comprehensive service providers

**Source of referral for patients with established HCV diagnosis to the CSP**	% of CSPs*
• Primary care	41
• Prison	14
• DAT	24
• GUM	14
• Hepatology	0
• Gastroenterology	10
• ID	5
• Other	12

**Source of referral when HCV diagnosed by the CSP**	**% of CSPs**

• Primary care	73
• Prison	4
• DAT	14
• GUM	6
• Hepatology	3
• Gastroenterology	17
• ID	12
• Other	14

*Totals can exceed 100% as more than one response

**Table 3:** Eligibility for anti-viral treatment of patients with Hepatitis C and reasons for patient refusal

**Question**	**Choices**	**Percentage of units saying yes**
**Which of the following criteria do you consider in determining eligibility for treatment?**		
	Age	61
	Gender	8
	Genotype	29
	Severity of hepatitis	93
	Co-morbidities	95
	*73% current/future drug/alcohol use*	
		
**What were the main reasons for patients' ineligibility?**		
	Ongoing drug use	62
	Ongoing alcohol use	21
	Psychiatric disorder	11
	Other medical co-morbidities	3
	Cardiovascular disease	2
		
**What are main reasons for patient refusal of treatment?**		
	Concern over side effects	49
	Inconvenient to start treatment -work pressures	37
	Refusal to modify chaotic lifestyle	30
	Lack of belief of efficacy	15

**Table 4:** Pattern of drug prescribing by CSP (2001)

**Drug regime used by CSP**	**Interferon alone**	**Interferon & Ribavirin**	**Pegylated Interferon & ribavirin**	**Pegylated interferon alone**
% of patients receiving a particular drug regime as a % of overall prescribing by CSP* (Median)	**0–5**	**25–49**	**50–74**	**0–5**
**Number of CSPs using drug regime**	20	64	57	24

**Table 5:** Barriers to providing a high quality service

**Reason**	**Agree/strongly agree%**	**Unsure %**	**Disagree /strongly disagree %**
**Clinic waiting times**	58	3	39
**Biopsy waiting time**	45	3	52
**Staffing Capacity**	76	12	16
**Staffing Skill mix**	42	15	43
**Funding for Treatment**	72	4	24
**Patient Refusal**	32	19	49
**Patient non- attendance**	80	3	17
**Patient Identification**	31	31	29

## References

[B1] World Health Organisation (2002). Hepatitis C -global prevalence -update. Wkly epidemiol rec.

[B2] Marcellin P, Asselah T, Boyer N (2002). Fibrosis and disease progression in hepatitis C. Hepatology.

[B3] Manns MP, McHutchison JG, Gordon SC, Rustgi VK, Shiffman M, Reindollar R, Goodman ZD, Koury K, Ling M, Albrecht JK (2001). Peginterferon alfa-2b plus ribavirin compared with interferon alfa-2b plus ribavirin for initial treatment of chronic hepatitis C: a randomised trial. Lancet.

[B4] Fried MW, Shiffman ML, Reddy KR, Smith C, Marinos G, Goncales FL, Haussinger D, Diago M, Carosi G, Dhumeaux D, Craxi A, Lin A, Hoffman J, Yu J (2002). Peginterferon Alfa-2a plus Ribavirin for Chronic Hepatitis C Virus Infection. N Engl J Med.

[B5] National Institute for Clinical Excellence (2004). Interferon alfa (pegylated and non-pegylated) and ribavirin for the treatment of chronic Hepatitis C. TA75 London.

[B6] Scottish Medicines Consortium (2002). Pegylated interferon 2a and Hepatitis C in adults.

[B7] Department of Health E&W (2002). Hepatitis C strategy for England. London.

[B8] Department of Health E&W (2004). Hepatitis C Action Plan for England.

[B9] Scottish Needs Assessment Programme Office for Public Health in Scotland (2000). Hepatitis C. Glasgow.

[B10] Cohen S Managed Public Health Networks Turning theory into practice. http://www.fph.org.uk/policy%5Fcommunication/publications/.

[B11] British Liver Trust website. http://www.britishlivertrust.org.uk.

[B12] EASL International Consensus Conference on Hepatitis C (1999). Consensus Statement on Hepatitis C. Journal of Hepatology.

[B13] National Institute of Health Consensus Development Program (2002). Management of Hepatitis C.

[B14] Strader DB, Wright T, Thomas DL, Seef LB (2004). Diagnosis, Management, and Treatment of Hepatitis C. Hepatology.

[B15] Scottish Centre for Infection and Environmental Health http://www.show.scot.nhs.uk/scieh.

[B16] Health Protection Agency England & Wales http://www.hpa.org.uk/infections.

[B17] The British Liver Trust (2002). Hepatitis C – The Public Stealth Disease.

[B18] Department of Health E&W (2002). Getting Ahead of the Curve A strategy for combating infectious diseases. London.

[B19] Bird SM, Goldberg DJ, Hutchinson SJ (2001). Projecting severe sequelae of injection-related hepatitis C virus epidemic in the UK. Journal of Epidemiol & Biostatistics.

[B20] Law MG, Dore GJ, Bath N, Thompson S, Crofts N, Dolan K, Giles W, Gow P, Kaldor J, Loveday S, Powell E, Spencer J, Wodak A (2001). Modelling hepatitis c virus incidence, prevalence and long-term sequelae in Australia, 2001. International Journal of Epidemiology.

[B21] Zou S, Tepper M, El Saadany S (2000). Prediction of hepatitis C burden in Canada. Can J Gastroenterol.

[B22] Buti M, San Miguel R, Brosa M, Cabases JM, Medina M, Casado MA, Fosbrook L, Esteban R (2005). Estimating the impact of hepatitis C virus therapy on future liver-related morbidity, mortality and costs related to chronic hepatitis C. J Hepatol.

[B23] Parkes J, Roderick P, Bennett LLoyd B, Rosenberg WM (2003). Hepatitis C in the United Kingdom. A review of prevalence and service delivery. British Association for the Study of the Liver.

[B24] Hutchinson SJ, McIntyre PG, Molyneaux P, Cameron S, Burns S, Taylor A, Goldberg DJ (2002). Prevalence of hepatitis C among injectors in Scotland 1989–2000: declining trends among young injectors halt in the late 1990s. Epidemiol & Infect.

[B25] Hope V, Judd A, Hickman M, Lamagni T, Hunter G, Stimson G, Jones S, Donovan L, Parry J, Gill N (2001). Prevalence of Hepatitis C Among Injection Drug Users in England and Wales: Is Harm Reduction Working?. Am J Public Health.

[B26] Skipper C, Guy JM, Parkes J, Roderick P, Rosenberg WM (2003). Evaluation of a prison outreach clinic for the diagnosis and prevention of hepatitis C: implications for the national strategy. Gut.

[B27] Gore SM, Bird AC, Cameron SO, Hutchinson SJ, Burns SM, Goldberg DJ (1999). Prevalence of hepatitis C in prisons: WASH-C surveillance linked to self-reported risk behaviours. QJM.

[B28] Weild AR, Gill ON, Bennett D, Livingstone SJM, Parry JV, Curran L (2000). Prevalence of HIV, hepatitis B and hepatitis C antibodies in prisoners in England and Wales: a national survey. Commun Dis Public Health.

[B29] Champion JK, Taylor A, Hutchinson S, Cameron S, McMenamin J, Mitchell A, Goldberg D (2004). Incidence of hepatitis C virus infection and associated risk factors among Scottish prison inmates: a cohort study. Am J Epidemiol.

