# Supplementary material for: Variation in Hepatitis C services may lead to inequity of heath-care provision: a survey of the organisation and delivery of services in the United Kingdom
Source: BMC Public Health. 2006 Jan 10;6:3. doi: 10.1186/1471-2458-6-3 (PMC1382203; doi:10.1186/1471-2458-6-3)
Supplement: Additional File 1 — Questionnaire-For questionnaire used in the national survey. [file 1471-2458-6-3-S1.doc]

## Additional File 1 Questionnaire

## National Study of Hepatitis C Services

ID Region ID

Please complete the following details:

Name

Hospital

## Your role in management of Hepatitis C

1. What is your role in the management of Patients with Hepatitis C?

Tick answer that most applies to your practice.

1. I have no role in the management of patients with Hepatitis C.

*Please answer only Q10 on page 3 and return questionnaire in envelope provided.*

1. Diagnosis +/- initial investigations, followed by referral to a dedicated Hepatitis C service*.

*Please answer Q2-10 on pages 2-3 and return questionnaire in envelope provided.*

1. Provision of a dedicated Hepatitis C service*.

*Please proceed directly to Q11 on page 4.*

* Includes diagnosis, investigation, treatment, & follow-up

**Your replies will be treated in strict confidence**

# Your Hospital

1. What is the approximate size of the catchment population of your clinical practice?

100,000-300,000

300,001-500,000

500,001-1 million

>1 million

>2 million

>3 million

1. Please specify the description that best fits the population served by your clinical practice/hospital.

Urban (wholly)

Urban (predominantly)

Mixed urban (more urban than rural)

Mixed rural (more rural than urban)

Rural (predominantly)

Rural (wholly)

Other please specify

1. What proportions of patients diagnosed with Hepatitis C in your hospital trust are managed by the following?

Hepatologist* %

Gastroenterologist %

Infectious disease %

Genito urinary medicine %

Unsure

Other - please specify

# *(hepatologist = those doctors whose substantive work is in liver disease)

1. Approximately how many patients with Hepatitis C did you diagnose in 2001?

<5

6-9

10-19

20-29

30-39

40-49

>50

1. What percentage of these patients did you refer to a specialist Hepatitis C service?

0-4%

5-9%

10-24%

25-49%

50-74%

75-90%

>90

# Diagnosis

1. Please specify the scenario that best describes the setting of the majority of liver biopsies performed in your hospital for patients with Hepatitis C:

Unguided by you/SPR staff (day case)

Unguided by you/SPR staff (overnight stay)

Guided by you/SPR staff (day case)

Guided by you/SPR staff (overnight stay)

Guided by radiology team (day case)

Guided by radiology team (overnight stay)

Other - please specify

# Do you have access to the following tests?

| **Service** | **In house**  **(yes/no)** | **External**  **(yes/no)** | **Number of tests requested by you on average per month** |
| --- | --- | --- | --- |
| **Qualitative PCR** |  |  |  |
| **Viral load measurement** |  |  |  |
| **HCV genotyping** |  |  |  |
| **Specialist liver histopathology** |  |  |  |

1. Do you **ever** treat patients with Hepatitis C? Yes/No

**
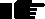
 If you do not provide a dedicated Hepatitis C service (Q1a), or have no role in the management of Hepatitis C (Q1b), please complete the following question.**

1. It would be very helpful to this national survey if you could state in the space below the name of the consultant who provides a specialist Hepatitis C service for your patient population.

Hospital _____________________________________________________________

# *Thank you very much for your help.*

# *Please return the questionnaire in the stamped addressed envelope provided. Contact details for queries are listed at the end of the questionnaire.*

Your replies will be treated in strict confidence.

**
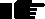
 If you provide a dedicated Hepatitis C service (Q1c), please complete the following questions.**

# Your Hospital

1. What is the approximate size of the catchment population in your practice with respect to the management of Hepatitis C?

100,000-300,000

300,001-500,000

500,001 – 1 million

>1 million

>2 million

>3 million

1. Please specify the description that best fits the population served by your clinical practice/hospital.

Urban (wholly)

Urban (predominantly)

Mixed urban (more urban than rural)

Mixed rural (more rural than urban)

Rural (predominantly)

Rural (wholly)

Other please specify

1. How many prisons are in your catchment* area? (*area from which regular referrals occur)

0

1

2

3

4

5

Don’t know

# Prevalence of Hepatitis C in your practice

1. What is the total number of patients with known hepatitis C currently under your care?
2. What was the approximate number of new patients seen by you in each of the following years?

|  | **1999** | **2000** | **2001** |
| --- | --- | --- | --- |
| **<10** |  |  |  |
| **10-19** |  |  |  |
| **20-34** |  |  |  |
| **35-49** |  |  |  |
| **50-74** |  |  |  |
| **75-100** |  |  |  |
| **>100 (please specify)** |  |  |  |

1. What is the approximate percentage of new patients who default from their initial out-patient appointment?

0-4%

5-9%

10-24%

25-49%

50-74%

>75%

1. What percentage of your time is spent on the clinical management of patients with Hepatitis C?

0-4%

5-9%

10-24%

25-49%

50-74%

>75%

## Referrals

# Source of referrals

*The following questions apply both to those patients referred to you in 2001 with a problem subsequently diagnosed as Hepatitis C AND also those referred to you with an established diagnosis of hepatitis C.*

1. What proportion of patients referred to you came with a diagnosis of Hepatitis C?

0-4%

5-9%

10-24%

25-49%

50-74%

>75%

Unsure

1. Where the ***initial*** diagnosis of hepatitis C was made before referral, please give the approximate percentage of referrals from each of these sources.

Primary care %

Prison healthcare %

Drug and Alcohol service %

Hepatology %

Gastroenterology %

Infectious diseases %

Genito urinary medicine %

Other please specify %

1. In those patients in whom YOU make the diagnosis of Hepatitis C, please indicate the source of referral with approximate proportions.

Primary care %

Prison healthcare %

Drug and Alcohol service %

Gastroenterology %

Infectious diseases %

Genito urinary medicine %

Hepatology %

Other - please specify

Unsure

# Management of patients with Hepatitis C

1. Do you ever refer patients with Hepatitis C to colleagues for further opinion or management?

Yes/No

If Yes

1. In what circumstances do you refer to colleagues?

For treatment

For follow up

For complex clinical issues relating to Hepatitis C

For joint management e.g. with psychiatry

For transplantation

Other - please specify

# Diagnosis

# Do you have the following investigative tests available?

| **Service** | **In house**  **(yes/no)** | **External**  **(yes/no)** | **Number of tests per month** |
| --- | --- | --- | --- |
| **Qualitative PCR** |  |  |  |
| **Viral load measurement** |  |  |  |
| **HCV genotyping** |  |  |  |
| **Specialist liver histopathology** |  |  |  |

# Liver Biopsy

1. Please specify the scenario that best describes the setting of the majority of liver biopsies performed in your hospital for patients with Hepatitis C.

Unguided by you/SPR staff (day case)

Unguided by you/SPR staff (overnight stay)

Guided by you/SPR staff (day case)

Guided by you/SPR staff (overnight stay)

Guided by radiology team (day case)

Guided by radiology team (overnight stay)

Other - please specify

# Counselling

1. What counselling and support services are available in your practice/hospital for patients with Hepatitis C?

Access to HCV specialist (any profession/grade) for pre and post-test counselling and support

Access to general counselling services

No access to counselling services

Other - please specify

## Treatment

# Initiating treatment

1. Which of the following criteria do you consider in determining eligibility for treatment?

Age Yes/No

Gender Yes/No

HCV Genotype Yes/No

Severity of hepatitis Yes/No

Co-morbidities Yes/No

Other - please specify Yes/No

1. Would you be likely to offer treatment to a patient with the following scenarios who had no additional contraindications:

Mild hepatitis on biopsy Yes/No

*If yes*: Asymptomatic Yes/No

With symptoms Yes/No

Moderate hepatitis Yes/No

Severe hepatitis Yes/No

Cirrhosis

Child - Pugh A Yes/No

Child - Pugh B Yes/No

Child - Pugh C Yes/No

Patient awaiting transplantation Yes/No

1. Which of the following patients with moderate/severe Chronic Hepatitis C are likely to receive treatment in your clinical practice? Please tick all that apply.

Continuing injecting drug user who regularly uses needle exchange Yes/No

Ex-injecting drug user stable on substitution therapy Yes/No

(If yes, for how long must they have been stable on substitution?)

Heavy alcohol consumer in regular employment Yes/No

17 year old person with haemophilia Yes/No

38 year old person with haemophilia without biopsy Yes/No

Person currently in treatment for psychiatric problems Yes/No

Persons with past history of parasuicide

whilst using drugs of addiction Yes/No

in context of previous non drug related psychiatric problems

Yes/No

Person with current diagnosis of depression related to HCV infection

Yes/No

Person with current diagnosis of depression unrelated to HCV infection

Yes/No

Person with past history of depression Yes/No

Person with poorly controlled hypertension Yes/No

Person with poorly controlled angina Yes/No

1. What proportion of new patients with Hepatitis C seen in your clinical practice in 2001 were **eligible** for treatment?

0-5%

6-9%

10-24%

25-49%

50-74%

75 -89%

>90%

1. What were the main reasons for patients’ ineligibility? Please rank 1-6 in descending order of importance.

Psychiatric disorder

Cardiovascular disease

Ongoing illicit drug misuse

Ongoing alcohol misuse

Other medical co-morbidities

Other (Please specify)

1. What proportion of your patients with Hepatitis C eligible for treatment received treatment in 2001?

0-5%

6-9%

10-24%

25-49%

50-74%

75 -89%

>90%

1. How many of these have been treated in the context of clinical trials?

0-5%

6-9%

10-24%

25-49%

50-74%

75 -89%

>90%

1. What antiviral drug regimes do you currently use to treat patients with Hepatitis C? Please tick all that apply.

Interferon alpha alone

Interferon alpha and ribavirin

Pegylated interferon alone

Pegylated interferon and ribavarin

Amantadine

‘Alternative’ medicines such as milk thistle

Other - please specify

1. What percentage of the patients you treated received the following treatment regimes in 2001?

|  | **0-5%** | **6-9%** | **10-24%** | **25-49%** | **50-74%** | **75-89%** | **>90%** |
| --- | --- | --- | --- | --- | --- | --- | --- |
| **interferon alone** |  |  |  |  |  |  |  |
| **interferon and ribavirin** |  |  |  |  |  |  |  |
| **interferon alone followed by combination therapy** |  |  |  |  |  |  |  |
| **Pegylated interferon alone** |  |  |  |  |  |  |  |
| **Pegylated interferon + ribavarin** |  |  |  |  |  |  |  |
| **Other - please specify** |  |  |  |  |  |  |  |

# Stopping treatment

1. Which of the following criteria do you use to end treatment? Please tick all appropriate boxes.

| **Therapy regime** | **HCV PCR +** | | **Persistently raised ALT** | | **Lack of reduction in viral load** | |
| --- | --- | --- | --- | --- | --- | --- |
|  | 3 months | 6 months | 3 months | 6 months | 3 months | 6months |
| **IFN alone** |  |  |  |  |  |  |
| **IFN/R** |  |  |  |  |  |  |
| **Peg IFN** |  |  |  |  |  |  |
| **Peg IFN/R** |  |  |  |  |  |  |

1. What treatment is offered to patients who do not respond/relapse on initial treatment? Please tick all appropriate boxes.

| **First Treatment** | **IFN**  ***R*** | **IFN**  ***NR*** | **IFN/Rib**  ***R*** | **IFN/Rib *NR*** | **Peg IFN** R | **Peg IFN** NR | *Peg IFN/Rib*R | *Peg IFN/Rib*NR |
| --- | --- | --- | --- | --- | --- | --- | --- | --- |
| **Second Treatment drug** |  |  |  |  |  |  |  |  |
| **Length of this treatment**  **(mths)** |  |  |  |  |  |  |  |  |

IFN = interferon Rib = ribavarin***NR =*** non-responderPeg IFN = pegylated interferon ***RR* =** relapsed responder

1. Do you have printed guidelines for dose reduction and stopping therapy?

Yes/No

If yes, we would be grateful for a copy to be enclosed with your returned questionnaire.

1. In what percentage of patients do you perform a post treatment liver biopsy?

0-5%

6-9%

10-24%

25-49%

50-74%

75-89%

>90%

# Refusal of treatment

1. What reasons do eligible patients who refuse treatment give? Please tick all that apply.

Refusal to modify chaotic lifestyle

Lack of belief in treatment effectiveness

Concern over adverse drug reactions

Inconvenient to start treatment due to work pressures

Lack of concern over future

Other - please specify

1. What percentage of patients stop treatment prematurely (both patient and professional initiated)?

0-5%

6-9%

10-24%

25-49%

50-74%

75 -89%

>90%

1. What are the reasons for stopping treatment prematurely? Please rank 1-5 in descending order of importance.

No response to treatment

Side effects (patient initiated)

Side effects (professional initiated)

Loss to follow up

Other - please specify

## Monitoring

*The following questions refer to the way in which you monitor patients with Hepatitis C.*

1. For patients who **ARE** receiving treatment, which members of your team sees them at follow-up visits once treatment has started? Tick all that apply.

| Staff | **Week 1-2** | **Week 3 – end month 3** | **Month 4 – end of treatment** | **Post Treatment** |
| --- | --- | --- | --- | --- |
| **Consultant, senior lecturer or professor** |  |  |  |  |
| **Specialist Nurse** |  |  |  |  |
| **Associate specialist/**  **staff grade** |  |  |  |  |
| **SPR (NHS)** |  |  |  |  |
| **SPR (R&D)** |  |  |  |  |
| **Other**  **Please specify** |  |  |  |  |

1. For patients with Hepatitis C **NOT** receiving treatment, who usually sees them for follow up? Tick all that apply.

| **Consultant, senior lecturer or professor** |  |
| --- | --- |
| **Specialist Nurse** |  |
| **Associate specialist/staff grade** |  |
| **SPR (NHS)** |  |
| **SPR (R&D)** |  |
| **Other (please specify)** |  |

1. For patients with Hepatitis C NOT receiving treatment, how often are they seen for follow up?

|  | **Mild hepatitis** | **Moderate hepatitis** | **Severe hepatitis** | **Cirrhosis** |
| --- | --- | --- | --- | --- |
| **Weekly** |  |  |  |  |
| **Monthly** |  |  |  |  |
| **Every 3 months** |  |  |  |  |
| **Every 6 months** |  |  |  |  |
| **Annually** |  |  |  |  |
| **Other (please specify)** |  |  |  |  |

## Service configuration

# Staffing

1. Please indicate which and how many at each grade of staff manage patients with Hepatitis C at your hospital. (Write approximate numbers of whole time equivalent staff in the appropriate boxes).

|  | **Consultant, Senior Lect or Professor** | **Associate specialist or Staff grade** | **SPR NHS** | **SPR R&D** | **Specialist nurse NHS*** | **Specialist nurse R&D** |
| --- | --- | --- | --- | --- | --- | --- |
| Hepatologist |  |  |  |  |  |  |
| **Gastroenterologist** |  |  |  |  |  |  |
| **Infectious disease** |  |  |  |  |  |  |
| **Genito urinary medicine** |  |  |  |  |  |  |
| Other |  |  |  |  |  |  |

* major part of their work is with patients with Hepatitis C

# Facilities

1. How many patients under your care for Hepatitis C were admitted as in-patients in 2001 for the following:

Liver biopsy

Complications of Hepatitis C

Non-liver related episode

Other

Unsure

1. Do you have any Out Reach Clinics for patients with Hepatitis C?

Yes/No

1. If Yes, please specify in the table details of any out reach services.

| **Location of out reach service** | **Specialist*** | **General**** |
| --- | --- | --- |
| **Primary Care (no of practices visited)** |  |  |
| **Homeless Units** |  |  |
| **Prison**  **(no of prisons visited)** |  |  |
| **Genito urinary clinics** |  |  |
| **Drugs and alcohol service** |  |  |
| **Other please specify** |  |  |

*most patients have Hepatitis C

**most patients come from broad remit of your speciality

1. We are interested the management of local outreach services and would be grateful for details of models in your region. Please give details if this applies to you (use additional sheet if necessary or append any documentation that explains the operation of your clinics, as appropriate).

1. What happens to clinic non-attenders?

New appointment sent once

New appointment sent twice if default once

Follow up by staff

Letter to GP

No action

Specific initiatives to improve attendance please specify below

Other - please specify

# Case finding (Testing for HCV in population at high risk)

1. Is case finding performed for Hepatitis C patients in your catchment population, excluding blood donors?

Yes/No/Unsure

If Yes

1. Is this performed according to a written policy?

Yes/No

If yes, we would be grateful for a copy to be enclosed with your returned questionnaire.

1. Which population is being tested? Tick all that apply.

Patients with established liver disease

Patients with abnormal LFTs in secondary/tertiary care

Patients with abnormal LFTs in primary care

Intravenous drug users in secondary/tertiary care

Intravenous drug users in primary care

Ex Intravenous Drug Users

Prisoners in your catchment area

Homeless population

High risk medical/surgical patients eg renal patients

Other - please specify

Unsure

# Liaison

1. Do you have a coordinated management strategy for patients with HCV linking secondary/tertiary care to:

Primary care Yes/No

Prison healthcare Yes/No

Drugs and alcohol services Yes/No

Homeless Yes/No

Genito Urinary services Yes/No

Other - please specify

If Yes to any of the above

1. Please state briefly a summary of the strategy or attach documentation if available.
2. Do you have a multidisciplinary team that coordinates the management of Hepatitis C in your area?

Yes/No/Unsure

If Yes:

1. Please indicate the membership of the group. Tick all that apply.

Hepatologist

Specialist nurse

Histopathologist

Radiologist

Infectious disease clinician

Community Drug and Alcohol Team representative

Care professional from homeless agency

Genito urinary medicine clinician

Community Intravenous drug users care professional

Patient representative

Community dentist

Other - please specify

# Record and databases

1. Do you have a database of patients with hepatitis C?

Yes/No/Unsure

If Yes

1. Is this Paper based Yes/No

Electronic Yes/No

Other - please specify Yes/No

1. What do you record in the database?

Patient demographics

Source of referral

Date of onset of hepatitis C diagnosis

Likely source of infection

Diagnostic test performed

Liver pathology

Treatment regime

Other - please specify

1. How long have you kept a record of patients with hepatitis C?

# Barriers and blockage in the management of patients with Hepatitis C

1. Are there any identified barriers in the management of patients with Hepatitis C?

Yes/No

If Yes:

1. Please indicate your response to each of the given statements describing possible barriers to care for patients with Hepatitis C.

|  | **Stongly agree** | **Agree** | **Unsure** | **Disagree** | **Strongly disagree** |
| --- | --- | --- | --- | --- | --- |
| **Clinic waiting time for initial referral** |  |  |  |  |  |
| **Biopsy waiting times** |  |  |  |  |  |
| **Staffing capacity** |  |  |  |  |  |
| **Staffing skillmix** |  |  |  |  |  |
| **Funding for treatment** |  |  |  |  |  |
| **Patient refusal** |  |  |  |  |  |
| **Patient non attendence** |  |  |  |  |  |
| **Patient identification** |  |  |  |  |  |
| **Other - please specify** |  |  |  |  |  |

Please write any additional comments below

64. Please give the approximate numbers of patients with Hepatitis C who are currently

Awaiting out patient appointment

Awaiting investigations

Awaiting funding decisions

Awaiting treatment

Other points in the health/social care systems where patients are waiting (please specify)

65. If you would like to make any further comments on aspects of care for patients with Hepatitis C, please use the space below.

If you would be willing to receive a telephone interview and perhaps a site visit from our researcher to your unit, please indicate below. This would be especially appreciated if you have novel models of care, databases or exciting initiatives in the management of patients with Hepatitis C

Yes/No

Contact telephone number:

Thank you very much indeed for taking the time to fill this questionnaire. It will make a substantial contribution to the completeness of the national needs assessment of Hepatitis C in UK and provide information with which to plan future services.
